# The impact of the COVID-19 pandemic on administrative eating disorder prevalence in the outpatient sector and on severity of anorexia nervosa

**DOI:** 10.1007/s00787-024-02527-2

**Published:** 2024-07-20

**Authors:** Friederike I. Tam, Richard Ochmann, Jörg Marschall, Henri Leschzinski, Maria Seidel, Linda Klink, Manuel Föcker, Katharina Bühren, Brigitte Dahmen, Charlotte Jaite, Beate Herpertz-Dahlmann, Jochen Seitz, Susanne Gilsbach, Christoph U. Correll, Antonia E. Müller, Johannes Hebebrand, Rebecca Bell, Tanja Legenbauer, Martin Holtmann, Katja Becker, Linda Weber, Marcel Romanos, Karin Egberts, Michael Kaess, Christian Fleischhaker, Eva Möhler, Ida Wessing, Daniela Hagmann, Freia Hahn, Ulf Thiemann, Gisela Antony, Katrin Gramatke, Veit Roessner, Stefan Ehrlich

**Affiliations:** 1 Division of Psychological and Social Medicine and Developmental Neurosciences, Faculty of Medicine, Technische Universität Dresden, 01307 Fetscherstraße 74, Dresden, Germany; 2 Eating Disorder Treatment and Research Center, Department of Child and Adolescent Psychiatry, Faculty of Medicine, Technische Universität Dresden, Dresden, Germany; 3 IGES Institute GmbH, Berlin, Germany; 4 LWL University Hospital Hamm for Child and Adolescent Psychiatry, Psychotherapy and Psychosomatics, Ruhr University Bochum, Hamm, Germany; 5 Department of Child and Adolescent Psychiatry, University Hospital Münster, Münster, Germany; 6 kbo-Heckscher-Klinikum, Academic Teaching Hospital of the LMU University, Munich, Germany; 7 Department of Child and Adolescent Psychiatry, Psychosomatics and Psychotherapy, RWTH Aachen University, Aachen, Germany; 8 Department of Child and Adolescent Psychiatry, Psychosomatic Medicine and Psychotherapy, Charité-Universitätsmedizin Berlin, corporate member of Freie Universität Berlin, Humboldt Universität zu Berlin, and Berlin Institute of Health, Berlin, Germany; 9 Department of Clinical Psychology and Psychotherapy in Childhood and Adolescence, University of Hildesheim, Hildesheim, Germany; 10 Department of Psychiatry and Molecular Medicine, Donald and Barbara Zucker School of Medicine at Hofstra/Northwell, Hempstead, NY USA; 11 German Center for Mental Health (DZPG), partner site Berlin, Berlin, Germany; 12 Department of Child and Adolescent Psychiatry, Psychosomatics and Psychotherapy, University Hospital Essen (AöR), University of Duisburg-Essen, Essen, Germany; 13 Department of Child and Adolescent Psychiatry, Psychosomatics and Psychotherapy, University of Marburg & University Hospital Marburg (UKGM), Marburg, Germany; 14 Department of Child and Adolescent Psychiatry, Psychosomatics and Psychotherapy, University Hospital Wuerzburg, Wuerzburg, Germany; 15 University Hospital of Child and Adolescent Psychiatry and Psychotherapy, University of Bern, Bern, Switzerland; 16 Department of Child and Adolescent Psychiatry and Psychotherapy, University Freiburg, Freiburg, Germany; 17 Department of Child and Adolescent Psychiatry, Saarland University, Homburg, Germany; 18 Department of Child and Adolescent Psychiatry, Psychosomatics and Psychotherapy, University of Tübingen, Tübingen, Germany; 19 Department of Child & Adolescent Psychiatry and Psychotherapy, LVR Hospital Viersen, Viersen, Germany; 20 Department of Child and Adolescent Psychiatry, Psychosomatics and Psychotherapy, LVR Hospital Bonn, Bonn, Germany; 21 Central Information Office, CIO Marburg GmbH, Fronhausen, Germany; 22 Department of Child and Adolescent Psychiatry, Faculty of Medicine, Technische Universität Dresden, Dresden, Germany

**Keywords:** COVID-19 pandemic, Eating disorders, Anorexia nervosa, Disorder severity, Healthcare research, Secondary data analysis

## Abstract

The COVID-19 pandemic appears to have had a considerable impact on the mental health of children and adolescents, particularly regarding eating disorders. However, it remains unclear whether the pandemic affected only the frequency or also the severity of eating disorders. We examined potential pandemic-related changes in the administrative prevalence of eating disorders in the outpatient sector compared with other mental disorders using German statutory health insurance data for the age group 10 to 16 years. We also examined disorder severity of anorexia nervosa using data from the multicenter German Registry of Children and Adolescents with Anorexia Nervosa in the same age group. Our results showed a marked increase in the administrative prevalence of eating disorders (based on documented diagnoses) in the outpatient sector among girls but not among boys. A similar pattern was found for internalizing disorders, whereas the administrative prevalences of externalizing disorders decreased. Regarding the severity of anorexia nervosa among inpatients, we found no pandemic-related changes in body mass index standard deviation score at admission, body weight loss before admission, psychiatric comorbidities and psychopharmacological medication. Given the administrative prevalence increase in the outpatient sector, the lack of impact of the pandemic on the inpatient sector may also be partly due to a shift in healthcare utilization towards outpatient services during the pandemic. Thus, the higher number of children and adolescents requiring specialized and timely outpatient care may be a major concern under pandemic conditions.

**Supplementary Information:**

The online version contains supplementary material available at 10.1007/s00787-024-02527-2.

## Introduction

A growing body of research around the world has been examining the impact of the COVID-19 pandemic on the prevalence and severity of mental disorders, with heterogenous results in adult populations [1, 2]. Less is known about the impact of the pandemic on the mental health of children and adolescents, who faced unique challenges such as school closures and distance learning, as well as isolation from peers [3]. In a representative German survey of children and adolescents aged 7 to 17 years, 71% reported feeling burdened by the pandemic, and the majority of participants found learning more difficult and had fewer social contacts than before the pandemic [4]. In addition, during the lockdown, children spent less time outdoors and had higher screen time [5, 6]. A recent systematic review, that included participants up to the age of 24 years, found that most studies indicated deteriorating mental health during the pandemic [3].

Eating disorders may be among the mental disorders experiencing the largest pandemic-related effects. A recent meta-analysis showed a 30% increase in the prevalence of eating disorders in a large pooled sample of mixed age [7]. Among eating disorders, the increase in incidence appeared to be most pronounced for anorexia nervosa (AN) [8]. Furthermore, there seem to be substantial sex/gender differences. For instance, a population-based study from the United Kingdom reported a pandemic-related increase in the number of first-time diagnoses of an eating disorder among females aged 10 to 24 years compared with pre-pandemic levels but a decrease among males [9]. However, while measures of disorder frequency suggest considerable pandemic-related effects [8], it is unclear whether disorder severity has also been affected. A cross-sectional study carried out during the pandemic found higher eating-related concerns and general psychological distress for adolescents with eating disorders in comparison to their unaffected siblings and to adults with eating disorders [10]. It is possible that patients who developed AN or relapsed during the pandemic also had more severe symptoms, such as being more severely underweight or experiencing greater weight loss, having more comorbidities or being more likely to require psychopharmacological medication.

The aim of this study was to examine the influence of the COVID-19 pandemic on (A) the administrative prevalence of eating disorders (based on documented diagnoses) compared with other mental disorders in the outpatient sector using routine healthcare data, and (B) on the severity of AN in inpatients using registry data, differentiated by sex and age. Overall, large-scale studies exploring pandemic-related effects on children’s and adolescents’ mental health are still rare, especially in the field of eating disorders. Our approach addresses this knowledge gap by combining information from two large and well-defined datasets that complement each other.

## Methods

### Datasets and measures

A) Administrative prevalences: We used serial cross-sectional data from children and adolescents aged 10 to 16 years with statutory health insurance living in the German federal state of Saxony to assess the administrative prevalence (based on documented diagnoses) of eating disorders and other mental disorders in the outpatient sector before and during the COVID-19 pandemic. These routine data from the Association of Statutory Health Insurance Physicians in Saxony (Kassenärztliche Vereinigung Sachsen) were provided by the Central Research Institute of Ambulatory Health Care in Germany (Zentralinstitut für die kassenärztliche Versorgung in Deutschland).

In Germany, the first COVID-19 lockdown started on March 22, 2020. Thus, the nine calendar quarters before the first COVID-19 lockdown (January 2018 to March 2020) were defined as “before the COVID-19 pandemic/pre-pandemic period” and the five subsequent calendar quarters (April 2020 to September 2021) were defined as “during the COVID-19 pandemic/pandemic period” for this study.

The target population for the routine data analysis were statutory health insured individuals in the age group 10 to 16 years living in Saxony. For each calendar quarter, all children and adolescents in the age group 10 to 16 years were included in the target population, resulting in a varying composition of the target population from one calendar quarter to the next. This serial cross-sectional approach was appropriate for this study, as the focus was on the aforementioned age group and not on a specific birth cohort. To be included in the dataset, an individual had to have had at least one contact with outpatient medical or psychotherapeutic care during the observation period from January 2018 to September 2021. Privately insured patients as well as patients exclusively treated at outpatient departments of psychiatry or child or adolescent psychiatry hospitals or as inpatients were not included, as billing was not conducted through the Association of Statutory Health Insurance Physicians. Patients were identified when one of the target diagnoses was documented by the outpatient medical or psychotherapeutic practitioners (pediatricians, family doctors, child and adolescent psychiatrists, child and adolescent psychotherapists).

The numbers of individuals with a diagnosis or suspected diagnosis of eating disorders (ICD-10 code F50 including anorexia nervosa, atypical anorexia nervosa, bulimia nervosa, atypical bulimia nervosa, overeating associated with other psychological disturbances, vomiting associated with other psychological disturbances, other eating disorders, unspecified eating disorder), depressive episode/recurrent major depressive disorder (F32/F33), anxiety disorders (F40/F41), attention-deficit hyperactivity disorders (ADHD) (F90), and conduct disorders (F91) were assessed in each calendar quarter. For each calendar quarter, the number of individuals with at least one documented target diagnosis was determined. A diagnosis in one calendar quarter was sufficient to be identified as an individual with a target diagnosis (i.e., it was not necessary for the diagnosis to be made in multiple consecutive calendar quarters). An individual with documented diagnoses in multiple calendar quarters was identified as an individual with a target diagnosis for each of these calendar quarters.

To assess administrative prevalences of the diagnoses considered, the total target population, which consisted of all children and adolescents aged 10 to 16 years residing in Saxony who were insured in the statutory health insurance system (that is, excluding respective children in the private health insurance system), was estimated on the basis of the KM6 statutory health insurance member statistics. These annual statistics on insured persons, including status, age, place of residence and health insurance (abbreviated as KM6) are published by the Federal Ministry of Health (Bundesministerium für Gesundheit, see Supplement 1.1). For the years 2018 to 2020, a proportion of 89–90% (depending on the year) of children and adolescents in the target age group were estimated to be covered by statutory health insurance. The estimated total target population (statutory health insured individuals in the age group 10 to 16 years living in Saxony) has steadily increased over the observation period by around 2% compared to the previous year (year 2018: 207,872; year 2019: 212,022; year 2020: 216,113; year 2021: 220,566). The total target population was used as reference for the calculation of the prevalences of mental disorders. The mean of the prevalences in the calendar quarters before the first COVID-19 lockdown was compared to the mean of the prevalences in the calendar quarters during the COVID-19 pandemic to assess possible pandemic-related effects. Percentage prevalence changes were calculated according to the following formula:

$$\begin{aligned}& x=\\ &\quad \left(\frac{Mean\:prevalence\:during\:the\:COVID-19\:pandemic\:}{Mean\:prevalence\:before\:the\:COVID-19\:pandemic}-1\right) \\ &\quad *100\end{aligned}$$

To assess differences due to sex (based on information from health insurance providers) and age, the analyses were repeated separately for girls and boys in three different age groups (10–11 years, 12–14 years, 15–16 years). The summary metrics of this dataset without statistical metrics were previously published on the website of the Saxon State Ministry for Social Affairs and Social Cohesion in German [11].

B) Disorder severity: We used data from the multicenter German Registry of Children and Adolescents with AN for inpatients aged 10 to 16 years to assess markers of disorder severity for AN before and during the COVID-19 pandemic. This web-based clinical registry, which is one of the very few clinical registries worldwide to provide clinical data of patients with AN for research purposes [12, 13], includes standardized clinically assessed variables, which are rarely available in population-based datasets, such as anthropometric measures and information on comorbidities and medication. Sociodemographic and clinical data have been systematically collected at 17 study centers across Germany and one study center in Switzerland since January 2015 [12]. Primary outcomes were age-adjusted body mass index standard deviation score (BMI-SDS) at admission to inpatient treatment, body weight loss before admission, comorbid depressive, anxiety or obsessive-compulsive disorders, and psychopharmacological medication. Secondary outcomes were duration from first weight loss to admission to first inpatient stay and body weight gain during the inpatient stay.

Included patients were admitted to inpatient programs of one of the 18 participating child and adolescent psychiatric hospitals. All patients had received a diagnosis of AN or atypical AN according to DSM-5 from an expert clinician at the treatment center. Data on age, sex, comorbid somatic and psychiatric diagnoses and medication as well as body weight and body height were collected by the treating clinician. Patients and their families were asked about the onset of weight loss and initial body weight before weight loss to determine the duration from first weight loss to admission to first inpatient stay and body weight loss before admission. Data was transmitted anonymously to the internet-based German Registry of Children and Adolescents (further details are described in Bühren et al. [12] and Föcker et al. [13]).

The observation period was chosen similar to the outpatient sector dataset (admission to inpatient treatment before the first COVID-19 lockdown: January 2018 to March 2020, admission to inpatient treatment during the COVID-19 pandemic: April 2020 to September 2021), and analyses were also repeated differentiated by sex and age. The registry study received ethical approval from the local ethics committees of all centers, and all participants and their legal guardians gave written informed consent.

### Statistical analyses

A) Administrative prevalences: Wilson score 95% confidence intervals with finite population correction adjustment were calculated for the prevalences [14]. The Z-test for two proportions was used to test if the proportions of females and males differed between the pre-pandemic period and the pandemic period.

B) Disorder severity: All statistical analyses were performed using IBM SPSS Statistics for Windows, version 29 (IBM Corp., Armonk, NY). Group differences were tested between patients admitted to inpatient treatment before the first COVID-19 lockdown (January 2018 to March 2020) and patients admitted to inpatient treatment during the COVID-19 pandemic (April 2020 to September 2021). Due to deviations from normality in some variables, the non-parametric Mann-Whitney U test was used for BMI-SDS at admission, body weight loss before admission, time from first weight loss to admission to first inpatient stay, and body weight gain during inpatient stay. The χ²-test was used for comorbid depressive, anxiety or obsessive-compulsive disorder and psychopharmacological medication. Confidence intervals were calculated for all variables (Wilson score confidence intervals for proportions [14]). Based on the BMI, the age-corrected BMI-SDS was calculated [15, 16]. For the variables duration from first weight loss to admission to first inpatient stay and body weight loss before admission, only patients experiencing their first inpatient stay were included. Statistical significance was defined as *P* < 0.05. *P* values were adjusted for multiple comparisons using the False Discovery Rate (FDR) correction method of Benjamini and Hochberg [17].

## Results

### Administrative prevalences

**Fig. 1:**
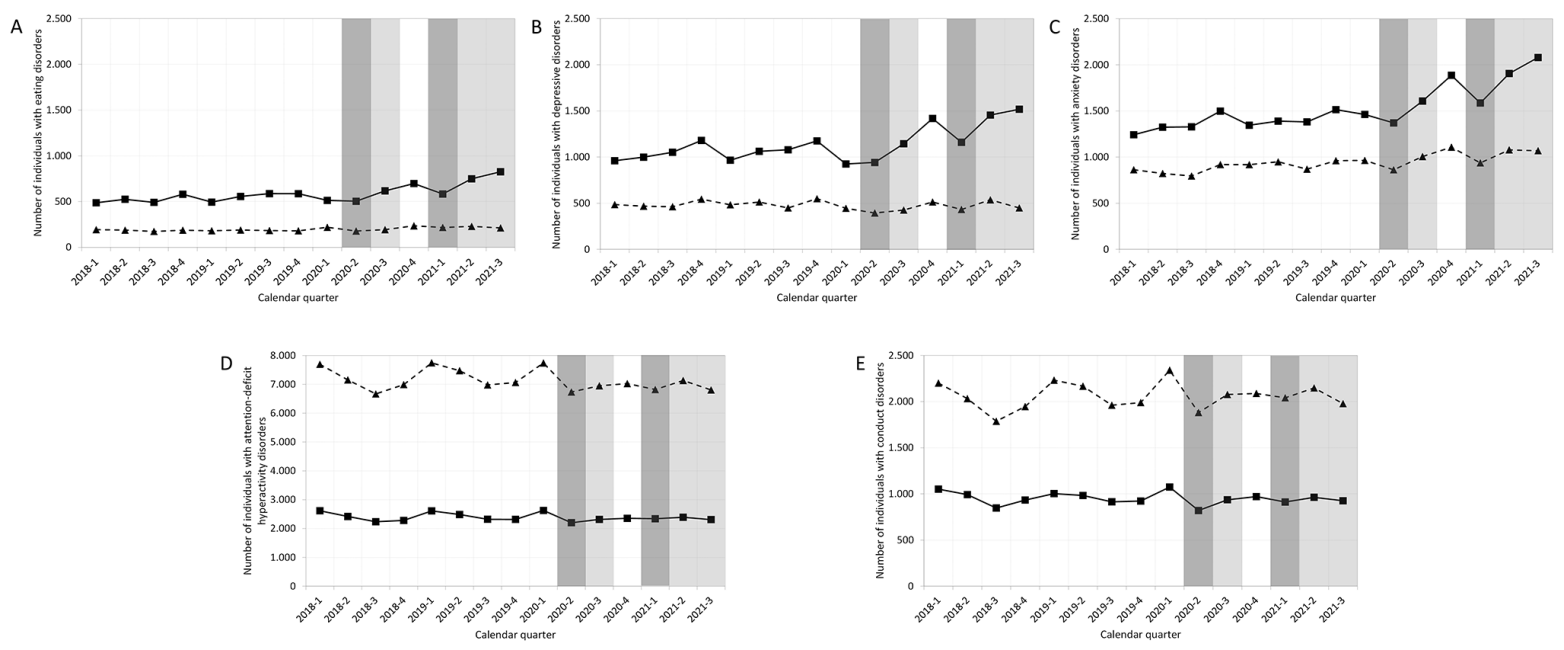
Number of outpatients aged 10 to 16 years with diagnoses or suspected diagnoses of different mental disorders over the observation period. Shown are numbers of girls (solid line, square marker) and boys (dashed line, triangle marker) before the first COVID-19 lockdown (calendar quarters 2018-1 to 2020-1, January 2018 to March 2020) and during the COVID-19 pandemic (calendar quarters 2020-2 to 2021-3, April 2020 to September 2021) with diagnoses or suspected diagnoses of (A) eating disorders, (B) depressive disorders, (C) anxiety disorders, (D) attention-deficit hyperactivity disorders and (E) conduct disorders. Darker shaded calendar quarters had strict restrictions on schools, including extended full closure. Lighter shaded calendar quarters did not have prolonged full school closures but did have restrictions such as limitations of class size and hygiene regulations

Comparing the pre-pandemic with the pandemic period, we observed an increase in the administrative prevalence of eating disorders in the outpatient sector by 20.2% (from 0.52 to 0.62%) among girls aged 10 to 16 years, whereas no change in administrative prevalence was observed among boys (see Table 1 for statistical details and population sizes). The female/male ratio was skewed towards girls, with no significant difference in the proportions of females and males with eating disorders before the COVID-19 pandemic compared to during the COVID-19 pandemic, 𝑧=−1.70, 𝑝=0.089. Focusing on age groups, no increase in eating disorder prevalence from the pre-pandemic to the pandemic period was observed among girls aged 10 to 11 years, while girls aged 12 to 14 years and girls aged 15 to 16 years experienced increases in prevalences of 15.2% (from 0.45 to 0.51%) and 28.3% (from 0.98 to 1.25%), respectively (Table 1). The absolute numbers of girls and boys with a diagnosis or suspected diagnosis of an eating disorder or another mental disorder for each calendar quarter are shown in Fig. 1 (see Supplement 2.1 for details on school-related restrictions).

A similar pattern was found for depressive disorders, with an increase in administrative prevalence of 18.4% (from 1.01 to 1.20%) for girls from the pre-pandemic to the pandemic period and no pandemic-related change in administrative prevalence for boys. The sex difference was less pronounced for anxiety disorders, with pandemic-associated prevalence increases of 21.8% (from 1.34 to 1.63%) for girls and 9.5% (from 0.82 to 0.90%) for boys. While the administrative prevalences of eating and internalizing disorders (depressive and anxiety disorders) increased during the COVID-19 pandemic compared to the pre-pandemic period, administrative prevalences of externalizing disorders (ADHD, conduct disorders) decreased slightly (based on healthcare utilization). Specifically, the prevalence of ADHD decreased by 7.5% (from 2.36 to 2.18%) for girls and 7.8% (from 6.70 to 6.17%) for boys, and the prevalence of conduct disorders decreased by 7.7% (from 0.94 to 0.87%) for girls and 4.7% (from 1.91 to 1.82%) for boys (Table 1).

### Disorder severity

During the observation period from January 2018 to September 2021, a total of 390 children and adolescents aged 10 to 16 years were included in the German Registry of Children and Adolescents with AN (treated in 14 out of the 18 participating child and adolescent psychiatric hospitals). Comparing the number of included patients per calendar quarter before and during the pandemic, there was no difference (before the COVID-19 pandemic: *median (interquartile range*,* IQR)* = 26.0 (5.0), during the COVID-19 pandemic = 27.5 (7.0), *U* = 33.50, *z* = 0.78, *P* = 0.456). Furthermore, there was no difference in age at admission to inpatient treatment before the pandemic (*median (IQR)* = 15.0 (1.9) years) in comparison to during the pandemic (*median (IQR)* = 15.0 (2.0) years, *U* = 19396.00, *z* = 0.79, *P* = 0.432).

Regarding the primary outcomes, there were no differences when comparing the pre-pandemic period with the pandemic period for BMI-SDS at admission, body weight loss before admission, prevalence of comorbid depressive, anxiety or obsessive-compulsive disorders, and use of psychopharmacological medication during the inpatient stay (Table 2). Furthermore, the time from first weight loss to admission to first inpatient stay did not change from the pre-pandemic period to the pandemic period (Table 2). Patients reached a similar body weight gain during the inpatient stay during the pandemic as before the pandemic (Table 2). For the analyses disaggregated by sex and age, statistical group comparisons were not performed for boys and for girls aged 10 to 11 years due to small sample sizes. For girls in the age groups 12 to 14 years and 15 to 16 years, no differences were found in any of the primary or secondary outcomes, mirroring the results from the full dataset.

**Table 1:** Administrative prevalences of mental disorders before and during the COVID-19 pandemic

Age (years)	10 to 11 years	12 to 14 years	15 to 16 years	Total
Population sizes Girls				
Before first COVID-19 lockdown During COVID-19 pandemic	30,714 31,615	44,109 45,338	28,516 29,446	103,339 106,399
Boys				
Before first COVID-19 lockdown During COVID-19 pandemic	32,463 33,471	46,188 47,686	30,012 30,783	108,663 111,940
Eating disorders Girls				
Before first COVID-19 lockdown (prevalence with 95%-CIs) During COVID-19 pandemic (prevalence with 95%-CIs) % prevalence change	0.19% (0.18, 0.21) 0.19% (0.18, 0.21) -2.2%	**0.45% (0.43**,** 0.47)** **0.51% (0.50**,** 0.53)** + 15.2%*	**0.98% (0.95**,** 1.01)** **1.25% (1.22**,** 1.30)** + 28.3%*	**0.52% (0.51**,** 0.53)** **0.62% (0.61**,** 0.64)** + 20.2%*
Boys				
Before first COVID-19 lockdown (prevalence with 95%-CIs) During COVID-19 pandemic (prevalence with 95%-CIs) % prevalence change	0.15% (0.14, 0.17) 0.16% (0.14, 0.17) + 0.4%	0.18% (0.17, 0.19) 0.20% (0.19, 0.22) + 12.4%	0.18% (0.17, 0.20) 0.20% (0.19, 0.22) + 10.5%	0.17% (0.17, 0.18) 0.19% (0.18, 0.20) + 8.6%
Depressive episode/recurrent major depressive disorder Girls				
Before first COVID-19 lockdown (prevalence with 95%-CIs) During COVID-19 pandemic (prevalence with 95%-CIs) % prevalence change	**0.15% (0.13**,** 0.16)** **0.18% (0.16**,** 0.19)** + 22.1%*	**0.74% (0.72**,** 0.77)** **0.84% (0.82**,** 0.87)** + 13.5%*	**2.36% (2.31**,** 2.41)** **2.83% (2.78**,** 2.89)** + 20.3%*	**1.01% (0.99**,** 1.03)** **1.20% (1.18**,** 1.22)** + 18.4%*
Boys				
Before first COVID-19 lockdown (prevalence with 95%-CIs) During COVID-19 pandemic (prevalence with 95%-CIs) % prevalence change	0.19% (0.18, 0.21) 0.18% (0.16, 0.19) -10.0%	0.42% (0.40, 0.44) 0.37% (0.36, 0.39) -11.4%	0.77% (0.74, 0.80) 0.72% (0.69, 0.75) -6.3%	0.45% (0.44, 0.46) 0.41% (0.40, 0.42) -8.9%
Anxiety disorders Girls				
Before first COVID-19 lockdown (prevalence with 95%-CIs) During COVID-19 pandemic (prevalence with 95%-CIs) % prevalence change	**0.96% (0.93**,** 0.99)** **1.09% (1.05**,** 1.12)** + 13.4%*	**1.16% (1.13**,** 1.19)** **1.41% (1.38**,** 1.45)** + 21.8%*	**2.04% (1.99**,** 2.08)** **2.56% (2.50**,** 2.62)** + 25.8%*	**1.34% (1.32**,** 1.36)** **1.63% (1.61**,** 1.66)** + 21.8%*
Boys				
Before first COVID-19 lockdown (prevalence with 95%-CIs) During COVID-19 pandemic (prevalence with 95%-CIs) % prevalence change	**0.81% (0.79**,** 0.84)** **0.87% (0.84**,** 0.90)** + 7.3%*	**0.79% (0.77**,** 0.81)** **0.91% (0.89**,** 0.94)** + 15.7%*	0.89% (0.86, 0.92) 0.92% (0.89, 0.95) + 3.1%	**0.82% (0.81**,** 0.84)** **0.90% (0.89**,** 0.92)** + 9.5%*
Attention-deficit hyperactivity disorders Girls				
Before first COVID-19 lockdown (prevalence with 95%-CIs) During COVID-19 pandemic (prevalence with 95%-CIs) % prevalence change	**3.03% (2.98**,** 3.08)** **2.67% (2.61**,** 2.72)** -12.0%*	2.26% (2.22, 2.31) 2.19% (2.15, 2.23) -3.3%	**1.78% (1.73**,** 1.82)** **1.64% (1.60**,** 1.69)** -7.5%*	**2.36% (2.33**,** 2.38)** **2.18% (2.15**,** 2.21)** -7.5%*
Boys				
Before first COVID-19 lockdown (prevalence with 95%-CIs) During COVID-19 pandemic (prevalence with 95%-CIs) % prevalence change	**7.57% (7.49**,** 7.64)** **6.89% (6.80**,** 6.98)** -9.0%*	**6.95% (6.88**,** 7.02)** **6.43% (6.36**,** 6.49)** -7.5%*	**5.37% (5.29**,** 5.45)** **5.01% (4.93**,** 5.08)** -6.8%*	**6.70% (6.66**,** 6.74)** **6.17% (6.13**,** 6.22)** -7.8%*
Conduct disorders Girls				
Before first COVID-19 lockdown (prevalence with 95%-CIs) During COVID-19 pandemic (prevalence with 95%-CIs) % prevalence change	1.06% (1.03, 1.10) 1.06% (1.02, 1.09) -0.6%	**0.93% (0.91**,** 0.96)** **0.84% (0.81**,** 0.86)** -10.3%*	**0.81% (0.78**,** 0.84)** **0.71% (0.68**,** 0.74)** -12.9%*	**0.94% (0.92**,** 0.96)** **0.87% (0.85**,** 0.88)** -7.7%*
Boys				
Before first COVID-19 lockdown (prevalence with 95%-CIs) During COVID-19 pandemic (prevalence with 95%-CIs) % prevalence change	**2.44% (2.40**,** 2.48)** **2.30% (2.25**,** 2.35)** -5.6%*	1.95% (1.91, 1.99) 1.88% (1.84, 1.92) -3.5%	1.27% (1.23, 1.31) 1.20% (1.16, 1.24) -5.7%	**1.91% (1.88**,** 1.93)** **1.82% (1.80**,** 1.84)** -4.7%*

Administrative prevalences of diagnoses or suspected diagnoses before the first COVID-19 lockdown (January 2018 to March 2020) and during the COVID-19 pandemic (April 2020 to September 2021) as well as percentage change in prevalence are shown for the total sample and separately for sex and different age groups. The reported population sizes were used as reference for the calculation of the prevalences. Wilson score 95% confidence intervals with finite population correction adjustment were calculated for all prevalences [14]. Statistically significant changes (95% confidence level, α = 0.05) are indicated in bold and marked with *.

**Table 2:** Markers of disorder severity and additional characteristics before and during the COVID-19 pandemic in female patients with anorexia nervosa or atypical anorexia nervosa

	*n*			12 to 14 years			15 to 16 years			Total		
	12 to 14 years	15 to 16 years	Total	Median/ Proportion (95%-CI)	Test statistics	*P*/*P*_adj_	Median/ Proportion (95%-CI)	Test statistics	*P*/*P*_adj_	Median/ Proportion (95%-CI)	Test statistics	*P*/*P*_adj_
**Primary outcomes: Disorder severity**												
BMI-SDS at admission												
Before COVID-19 lockdowns During COVID-19 pandemic	96 70	120 81	222 156	-2.27 (-2.66, -2.17) -2.33 (-2.67, -2.14)	*U =* 3363.50, *z =* 0.01	0.991/0.991	-2.54 (-2.75, -2.39) -2.63 (-2.79, -2.38)	*U* = 4707.00, *z*=-0.38	0.705/0.918	-2.41 (-2.63, -2.34) -2.45 (-2.66, -2.33)	*U* = 17036.50, *z*=-0.27	0.789/0.918
Body weight loss before admission in percent												
Before COVID-19 lockdowns During COVID-19 pandemic	64 38	72 53	141 94	21.6 (21.1, 25.1) 22.0 (19.2, 26.0)	*U* = 1187.00, *z*=-0.20	0.841/0.918	23.0 (20.1, 24.4) 23.7 (20.9, 25.6)	*U* = 1999.00, *z* = 0.46	0.649/0.918	22.7 (21.2, 24.0) 23.4 (20.9, 24.6)	*U* = 6712.50, *z* = 0.17	0.867/0.918
Comorbid depressive, anxiety or obsessive-compulsive disorder												
Before COVID-19 lockdowns During COVID-19 pandemic	96 67	120 74	222 146	34.4% (25.6, 44.3) 28.3% (19.0, 40.1)	χ²(1) = 0.66	0.417/0.918	37.5% (29.4, 46.4) 39.2% (28.9, 50.6)	χ²(1) = 0.06	0.814/0.918	35.6% (29.6, 42.1) 33.6% (26.4, 41.6)	χ²(1) = 0.16	0.690/0.918
Psychopharmacological medication												
Before COVID-19 lockdowns During COVID-19 pandemic	96 70	120 81	222 156	17.7% (11.4, 26.5) 27.1% (18.1, 38.5)	χ²(1) = 2.12	0.145/0.871	23.3% (16.7, 31.7) 25.9% (17.6, 36.4)	χ²(1) = 0.18	0.675/0.918	20.3% (15.5, 26.0) 25.6% (19.4, 33.0)	χ²(1) = 1.52	0.218/0.918
**Secondary outcomes: Additional patient characteristics**												
Time from first weight loss to admission to first inpatient stay in days												
Before COVID-19 lockdowns During COVID-19 pandemic	70 47	81 56	156 106	181 (162, 403) 213 (179, 378)	*U* = 1809.50, *z* = 0.92	0.360/0.918	212 (235, 349) 291 (132, 440)	*U* = 2695.50, *z* = 1.87	0.061/0.550	184 (221, 343) 245 (187, 369)	*U* = 9496.00, *z* = 2.04	0.041/0.550
Body weight gain during inpatient stay in percent												
Before COVID-19 lockdowns During COVID-19 pandemic	90 62	116 70	212 137	21.1 (19.7, 25.9) 20.1 (18.3, 25.3)	*U* = 2676.00, *z*=-0.43	0.669/0.918	19.5 (18.0, 22.6) 18.2 (16.3, 21.7)	*U* = 3854.50, *z*=-0.58	0.563/0.918	19.9 (19.5, 23.2) 19.3 (18.1, 22.3)	*U* = 13898.00, *z*=-0.68	0.498/0.918

For BMI-SDS at admission, body weight loss before admission, time from first weight loss to admission to first inpatient stay, and body weight gain during inpatient stay, the median and 95% confidence intervals for the mean were reported, and group differences were tested with the Mann-Whitney U test. For comorbid depressive, anxiety or obsessive-compulsive disorders and psychopharmacological medication, proportion and 95% confidence intervals (Wilson score intervals [14]) were reported, and group differences were tested with the χ²-test. Based on the BMI, the age-corrected BMI standard deviation score was calculated [15, 16]. *P* values were adjusted for multiple comparisons (*P*_*adj*_, six measures, three groups) using the False Discovery Rate (FDR) correction method of Benjamini and Hochberg [17]. *=*P*_*adj*_<0.05; **=*P*_*adj*_<0.01; ***=*P*_*adj*_<0.001. Regarding diagnostic assignment according to ICD-10, 269 female patients with anorexia nervosa (71%) were of the restrictive subtype, 49 (13%) were of the binge-purge subtype and 48 (13%) were diagnosed with atypical anorexia nervosa (subtype not reported in 3%). Abbreviations: BMI-SDS, body mass index standard deviation score; 95%-CI, 95% confidence interval.

## Discussion

For this publication, we investigated the influence of the COVID-19 pandemic on the administrative prevalence of eating disorders compared to other mental disorders in the outpatient sector as well as on disorder severity of AN in children and adolescents aged 10 to 16 years. Using routine data from the Association of Statutory Health Insurance Physicians in Saxony, we found a 20% pandemic-related increase in the administrative prevalence of eating disorders in the outpatient sector among girls, but not among boys. Among different age groups, girls aged 15 to 16 years experienced the highest increase in prevalence (28%). A similar pattern was found for internalizing disorders (pandemic-related prevalence increase of 18% for depressive disorders and of 22% for anxiety disorders), whereas externalizing disorders (ADHD, conduct disorders) decreased in prevalence. Using data from the German Registry of Children and Adolescents with AN, no pandemic-related changes were found for indicators of disorder severity of AN or additional patient characteristics in inpatients. To our knowledge, this study is the first to use clinical registry data to examine the impact of the COVID-19 pandemic on the severity of AN.

Our finding of an increase in the administrative prevalence of eating disorders is consistent with the results of a meta-analysis based on a large sample of different ages and eating disorder diagnoses [7]. In line with this, a Finnish national register-based study (including residents up to the age of 17 years) reported an increase in the incidence of eating disorders during the pandemic of 27% [18]. A study based on electronic health records of individuals up to the age of 30 years, mostly in the United States of America, reported eating disorder incidences to increase by 15% during the pandemic [8]. Also indicative of a prevalence increase of eating disorders, visits for eating disorders to pediatric primary care providers in Massachusetts (United States of America) doubled during the pandemic compared with pre-pandemic levels [19]. While some previous studies found that eating disorders had the largest pandemic-related effects compared with other mental disorders [18, 19], our data suggest comparable effects for anxiety and depressive disorders. Our finding of an increase in internalizing disorders (depressive and anxiety disorders) is consistent with previous results [18, 20]. There may be several reasons as to why the prevalences of eating disorders and internalizing disorders increased during the pandemic, especially among girls. Increased loneliness was found to be associated with higher depressive levels during the pandemic for both boys and girls, and an association between time spent on social media/video games and depressive symptoms emerged for girls [21]. Other stressors associated with depression and/or anxiety in girls during the pandemic were problems with online learning, stress or disorientation from not having a schedule, and lack of privacy/space [22]. Long COVID itself may also have played a role, as appetite problems, depression, and anxiety were increased in children with prior COVID-19 infection [23]. Specifically for eating disorders, patients self-reported an increase in following models/influencers on social media and using apps for weight loss as well as an increase in preoccupation with cooking recipes, mirror checking (possibly augmented by the “zoom effect”, which describes body checking and comparison behavior during video calls), and conflicts with parents due to eating [24]. These behaviors may exacerbate or prolong eating disorder symptoms, thereby increasing prevalence at the population level. Furthermore, adolescents with eating disorders self-reported elevated posttraumatic symptoms during the pandemic in an Italian cross-sectional study, suggesting a possible traumatic role of the pandemic in the life-trajectory of some patients [10].

Our administrative prevalence data for eating disorders in the outpatient sector indicate a sex difference with an increase in prevalence only for girls and not for boys. This aligns with findings from the aforementioned electronic health record study [8] and a population-based primary care study in the United Kingdom [9]. Supporting a similar pandemic-related effect in internalizing disorders, we also found a strong sex difference for depressive disorders (increase in prevalence in girls but none in boys) and anxiety disorders (increase in prevalence in girls and considerably smaller increase in boys). This was in agreement with results from the Finnish national register-based study [18]. In summary, our findings, as well as those of previous studies, suggest a greater pandemic-related increase in the frequency of eating disorders and internalizing disorders in girls than in boys. However, sex/gender differences in health care utilization and help-seeking behavior during the pandemic should be taken into consideration as they may have led to an underestimation of affected boys. Sex/gender has been shown to influence health care utilization and help-seeking behavior, with girls being generally more willing to use mental health services than boys [25]. This effect may have been exacerbated during the pandemic, as evidenced by North American studies on mental-health related emergency department use [26, 27].

Looking at different age groups, we found no pandemic-related increase in administrative eating disorder prevalence among girls aged 10 to 11 years but increases among those aged 12 to 14 years and 15 to 16 years. This is in partial accordance with the findings from the aforementioned electronic health record study (mostly in the United States) [8], that reported no increased relative risk for a new eating disorder diagnosis in the age group 0 to 9 years but a significantly increased risk for the age groups 10 to 14 years and 15 to 19 years. When comparing this pattern with internalizing disorders, all age groups in our study population showed pandemic-related effects for depressive and anxiety disorders. However, for anxiety disorders, prevalence increases were more pronounced in the older age groups. A nationally representative caregiver survey of children and adolescents up to the age of 17 years from the United States of America also reported that increasing age was associated with higher odds of having singular or comorbid depression and anxiety [28]. It seems that for eating disorders and anxiety disorders, older adolescents were more severely affected by the pandemic. This may be due to the greater importance of peers, fewer restrictions in internet use and higher pressure due to academic requirements.

Regarding externalizing disorders, our finding of a decrease in the administrative prevalence of ADHD is in line with data on ADHD medication use (as a proxy for ADHD prevalence), which was lower than expected from pre-pandemic levels in Germany for both 2020 and 2021 [29]. While our data pointed to a decrease in the prevalence of ADHD during the pandemic in both girls and boys, a study from the United States of America suggested a sex/gender difference, with the percentage of individuals receiving prescription stimulants increasing for females but decreasing for males in 2020 to 2021 for the age groups 10 to 14 years and 15 to 19 years [30]. In this context, it is possible that some patients benefited from a less stimulating environment at home compared to school and were less socially overwhelmed during lockdown. The literature on the impact of the pandemic on conduct disorders is still scarce. While we found a decrease of conduct disorders, the Finnish national register study reported no significant difference in the number of new diagnoses compared to levels predicted on the basis of previous years [18]. Another possible explanation for the observed prevalence decrease for ADHD and conduct disorders may be that affected individuals were less likely to experience conflicts with rules at home during distance learning than at school. Furthermore, teachers may have been less likely to notice externalizing symptoms and suggest the initiation of diagnostic steps during the remote setting.

While eating disorder frequency seems to have increased during the COVID-19 pandemic, potential pandemic-related effects on disorder severity are largely unknown. Meta-analytic findings in a sample of different ages and eating disorder diagnoses suggested an increase in AN symptom severity and, for eating disorders in general, an increase in comorbid depression and hospitalization rates during the pandemic [7]. However, in our clinical registry dataset from child and adolescent psychiatry hospitals across Germany, we found no change in BMI-SDS at admission to inpatient treatment for girls with AN or atypical AN, which is similar to findings from pediatric hospitals [31–33]. Furthermore, there was no pandemic-related change in body weight loss before admission, which corresponds to findings from an Italian pediatric department [32]. A Canadian pediatric multicenter study, which included newly diagnosed individuals with AN assessed in a tertiary care setting, reported a pandemic-related increase of percentage body weight loss [31]. However, the body weight loss was lower than in our study sample (pre-pandemic and during the pandemic), which might be explained by a lower mean disorder severity in the Canadian sample, which also included outpatients [31]. The prevalence of psychiatric comorbidities and the proportion of patients receiving psychopharmacological medication in our sample did not change during the pandemic, which is in accordance with the findings of two pediatric samples [33, 34]. On the other hand, an Italian retrospective study reported an increase in comorbid major depressive disorder and a more than twofold increase in the proportion of patients requiring treatment with psychotropic drugs [32]. Furthermore, our registry data do not suggest a pandemic-related increase in inpatient admissions in girls with AN or atypical AN in participating child and adolescent psychiatry hospitals during the pandemic. This may be due to a ceiling effect as specialized centers often have limited capacity to admit new patients [35]. This limits comparability with general or pediatric hospitalization data, which suggest an increase in hospitalizations for adolescent AN [31–33, 36, 37]. In case of specialized centers, waiting time may have increased, as reported by five out of six eating disorder centers in Europe [35]. Also suggesting an increased need for specialized inpatient care for patients with AN during the pandemic, an Italian retrospective study reported that the proportion of patients with AN admitted to a specialized eating disorder unit increased during the COVID-19 pandemic (while the proportion of patients with binge eating disorder decreased) [38]. Although not directly assessed in our study population, the time from first weight loss to admission to first inpatient stay may serve as a proxy for waiting time and did not change during the pandemic in our dataset. Given the increase in prevalence observed in the outpatient sector, the lack of impact of the pandemic on the inpatient sector may be partly due to a shift in health care utilization towards outpatient services during the pandemic. Furthermore, we did not observe pandemic-related changes in age at admission to inpatient treatment, which is consistent with two previous studies [32, 33]. In contrast, hospital admission data from a German statutory health insurance company showed a higher increase in overall hospital admissions for AN among girls aged up to 14 years (40%) than among girls aged 15 to 19 years (32%) [37]. Interestingly, in boys, the same study showed a considerable increase in overall hospital admissions among boys aged up to 14 years (69%) but no increase for boys aged 15 to 19 years [37].

There are some limitations which should be considered in relation to our findings. First, the outpatient dataset provides secondary data (based on healthcare utilization), which cannot be equated to primary prevalence data. Furthermore, the outpatient dataset did not include individuals exclusively treated at outpatient departments of psychiatry or child and adolescent psychiatry hospitals or as inpatients. This may have led to an underestimation of the prevalences of the mental disorders studied. Moreover, the outpatient dataset did not differentiate between different eating disorder diagnoses. The two separate datasets used in the analyses differed with respect to health insurance (outpatient dataset: statutory health insurance only, inpatient dataset: statutory and private health insurance) and region (outpatient dataset: Saxony only, inpatient dataset: nationwide). Both health insurance coverage and regional differences can influence health care utilization [39, 40], which may be a limiting factor when comparing the two datasets. Lastly, both datasets grouped patients binarily with respect to sex (female, male) and did not include information on transgender or non-binary identification, and no information on ethnicity was available.

In conclusion, our study provided evidence for an increase in the administrative prevalence of eating disorders in the outpatient sector among girls during the COVID-19 pandemic. We found a similar pattern for internalizing disorders, whereas the prevalence of externalizing disorders decreased. Data from the German Registry of Children and Adolescents with AN did not suggest pandemic-related changes in disorder severity or additional measures characterizing treatment in inpatients with AN. The observed lack of impact of the pandemic on the inpatient sector may also be partly due to a shift in healthcare utilization towards outpatient services during the pandemic. Thus, the higher number of children and adolescents requiring specialized and timely outpatient care may be a major concern under pandemic conditions.

## Electronic supplementary material

Below is the link to the electronic supplementary material.

Supplementary Material 1

## Data Availability

No datasets were generated or analysed during the current study.
