# Supplementary material for: The impact of the COVID-19 pandemic on administrative eating disorder prevalence in the outpatient sector and on severity of anorexia nervosa
Source: Eur Child Adolesc Psychiatry. 2024 Jul 20;34(3):983–95. doi: 10.1007/s00787-024-02527-2 (PMC11909050; doi:10.1007/s00787-024-02527-2)
Supplement: Supplementary file 1 — Supplementary Material 1 [file 787_2024_2527_MOESM1_ESM.docx]

***Supplementary Information for***

***The impact of the COVID-19 pandemic on administrative eating disorder prevalence in the outpatient sector and on severity of anorexia nervosa***

Correspondence to: Friederike Tam, Division of Psychological and Social Medicine and Developmental Neurosciences, Faculty of Medicine, Technische Universität Dresden, Fetscherstraße 74, 01307 Dresden, Germany, phone number: +49 351 458-5214, email: transden.lab@uniklinikum-dresden.de

**Supplement 1: Methods**

- 1. **Estimating of the total target population**

The total target population, which consisted of all children and adolescents in the age group of 10 to 16 years residing in Saxony and covered by statutory health insurance, was estimated using the KM6 statutory health insurance member statistics of the Federal Ministry of Health (Bundesministerium für Gesundheit). Data from the KM6 statutory health insurance member statistics were only available for the age group under 15 years in Saxony as of June 30 of a given year (until 2021). Thus, these data were based on a different age range than our target group (10 to 16 years). A comparison of the number of children and adolescents under 15 years of age from the KM6 statistics with the number of the corresponding age group in the population published by the Statistical Office of Saxony (Statistisches Landesamt in Sachsen) for the years 2018 to 2020 resulted in a proportion of 89% to 90% (depending on the year) of children and adolescents to be covered by statutory health insurance. Therefore, the same proportion was assumed for the age group 10 to 16 years and used to estimate the number of children and adolescents covered by statutory health insurance in this age group in Saxony from 2018 to 2021.

**Supplement 2: Results**

**2.1. School-related restrictions due to the COVID-19 pandemic in the German federal state of Saxony**

In April and May 2020, primary and secondary schools in Saxony were closed and replaced by distance learning. Exceptions were later made for final year students, some special education schools, and children of essential workers. From mid-May to August 2020, the COVID-19 incidence rate in Saxony decreased and schools reopened with smaller class sizes, alternating between distance learning and physical attendance. For grades 1-4, parents could choose between distance learning and physical attendance. As the public health situation improved, schools were able to largely return to physical attendance in September 2020, with limitations in class size and hygiene regulations. Between December 2020 and February 2021, rising COVID-19 incidence rates forced schools nationwide to close completely again (except for final year students). Starting in March 2021, depending on local infection rates, alternating physical attendance and distance learning or full distance learning was reintroduced, again with exceptions for final year students in primary and secondary schools.
